# A Novel Spectrophotometric Method for the Determination Oxacillin Sodium

**Published:** 2009-12

**Authors:** Rajinder Singh Gujral, Sk Manirul Haque, Prem Shanker

**Affiliations:** *Vardhman Chemtech Ltd, Nimbua, Dera Bassi, Mohali, Punjab, India*

**Keywords:** Oxacillin sodium, iodine, spectrophotometer, validation, human urine

## Abstract

A simple and sensitive spectrophotometric method was developed for the determination of Oxacillin sodium. The method was based on charge transfer complexation reaction of the drug with iodine in methanol – dichloromethane medium. The absorbance was measured at 365 nm against the reagent blank. Under optimized experimental conditions, Beer’s law is obeyed in the concentration ranges 2–8 μg/ml for Oxacillin Sodium. The method was validated for specificity, linearity, precision, accuracy, limit of quantitation, robustness and ruggedness. The LOD and LOQ value were 0.39 and 1.18 μg/ml respectively. The method was successfully applied to the analysis of Oxacillin sodium in Human Urine samples with good accuracy and precision.

## INTRODUCTION

**Figure 1:**
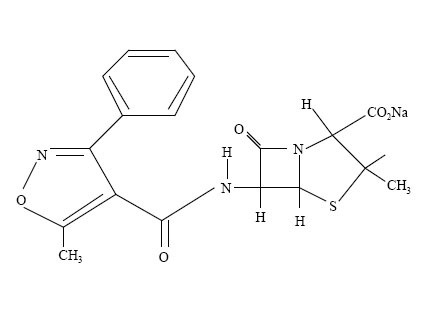
Structure of Oxacillin Sodium.

Oxacillin [(2S, 5R, 6R)-3, 3-dimethyl-6-[[(5-methyl l-3-phenylisoxazol-4-yl) carbonyl] amino]-7-oxo-4-thia-1-azabicyclo [3.2.0] heptane-2-carboxylate monohydrate] (Figure. 1) is the penicillinase-resistant penicillins.

Oxacillin sodium is semi synthetic penicillin. It is available commercially as the monohydrate sodium salt which occurs as a fine, white, crystalline powder that is odorless or has a slight odor. It is freely soluble in water and has a pK_a_ of about 2.8. It is resistant to acid inactivation in the gut, but is only partially absorbed after oral administration. The bioavailability after oral administration in humans has been reported to range from 30–35%, and, if given with food, both the rate and extent of absorption is decreased. In humans, approximately 89–94 % of the drug is bound to plasma proteins. It is partially metabolized to both active and inactive metabolites.

Numerous analytical methods have been reported in the scientific literature for the determination of oxacillin sodium. These methods are based on Spectrophotometric (1), ion exchange (2), photometric extraction (3), potentiometric (4) and high performance liquid chromatography (HPLC) (5–8). The determination in biological fluids normally requires the use of trace analysis techniques such as High Performance Liquid Chromatography (HPLC), Liquid Chromatography (LC), Capillary Electrophoresis (CE), Cyclic Voltametry, Liquid Chromatography-Mass Spectrophotometry (LC-MS), Gas Chromatography-Mass Spectrophotometry (GC-MS), Inductively Coupled Plasma-Mass Spectrophotometry. All of these methods are very expensive because it require long and tedious pretreatment of the samples and laborious clean up procedures prior to analysis.

Therefore, it is necessary to develop a simple and suitable analytical method for the determination of Oxacillin sodium. UV-Visible spectrophotometry is the technique of choice in research laboratories, hospitals and pharmaceutical industries due to its low cost and inherent simplicity.

The method was based on the charge transfer complexation of drug with iodine. The present method did not require laborious clean up procedures prior to analysis and absorbance was measured at 365 nm against the reagent blank prepared simultaneously. The method was also extended to the invitro determination of Oxacillin in human urine samples.

## EXPERIMENTAL

### Apparatus

Spectral runs were made on UV 3000^+^ UV-VIS Spectrophotometer (LABINDIA^®^, Mumbai, India) [Serial Number 17-1885-01-0016] with 1 cm matched glass cell.

### Materials and Reagents

- Oxacillin Sodium (Vardhman Chemtech Ltd, Mohali, Punjab, India) used as a working standard.
- Iodine (RFCL Limited, New Delhi, India) was prepared as 0.05% solution in dichloromethane.
- Sodium carbonate was purchased from Qualigens fine chemicals (Mumbai, India).
- Urine samples were obtained from healthy volunteers.
- Carbonate buffer of pH 9.4 was prepared in distilled water.

### Standard Oxacillin Solution

A stock solution of Oxacillin sodium (50 μg/ml) was prepared by dissolving 5 mg Oxacillin in 100 ml volumetric flasks with methanol. The stock solution (50 μg/ml) was used to prepare the working solutions by suitable dilutions with methanol. The solutions were stable at least 10 days at room temperature.

### Procedure for determination

Into a series 10 ml volumetric flasks, different volumes (0.04–0.16) ml of standard Oxacillin sodium (0.5 μg/ml) solution corresponding to 2.0–8.0 μg/ml were pipetted. To each flask, 1.5 ml of 0.05% iodine was added and diluted to volume with methanol. The reaction was allowed to proceed at room temperature and absorbance was measured as a function of time at 365 nm against reagent blank prepared simultaneously. The calibration curve was constructed by plotting the absorbance against the initial concentration of Oxacillin. The content of Oxacillin was calculated either from the calibration curve or corresponding regression equation.

### Procedure for determination of Oxacillin sodium in human urine samples

Aliquot volumes of oxacillin (50 μg/ml) were spiked with human urine samples and transferred into small separating funnel. 10 ml of carbonate buffer pH-9.4 was added and solution was mixed well. The solution was then extracted with 3 × 5 ml diethyl ether. The ether extract was collected and evaporated. The residue was dissolved in 15 ml distilled water and above general procedure was then followed. The amount of Oxacillin was obtained from the calibration graphs or corresponding regression equation.

## METHOD VALIDATION

Validation of analytical procedures is a vital aspect not just for regulatory purposes, but also for their efficient and reliable long-term application. In order to address the performance of the analytical procedure adequately, the analyst is responsible to identify the relevant parameters, to design the experimental validation studies accordingly and to define appropriate acceptance criteria.

### Solution Stability

The stability of oxacillin sodium quality control sample solutions at room temperature was evaluated with the help of UV-visible spectra

### Specificity and selectivity

The specificity and selectivity of the proposed method was evaluated by estimating the amount of oxacillin in the presence of common excipients such as sodium stearyl fumarate, magnesium stearate, starch, lactose, glucose, fructose and talc.

### Linearity

The linearity of the method was ascertained by taking oxacillin at nine concentration levels 2.0–8.0 μg/ml. Each concentration was independently analyzed five times.

### Accuracy and Precision

The accuracy and precision of the method was evaluated within the linear range based on the analysis of oxacillin standard samples at 2.0, 5.0 and 8.0 μg/ml. Five independent analysis were performed at each concentration level within one day (intra day precision) as well as for five consecutive days (inter day precision).

### Limit of detection (LOD)

$$\text{LOD}=3.3\times {\text{S}}_{\text{o}}/\text{b}$$

where S_O_ and b are standard deviation and slope of the calibration line, respectively.

### Limit of quantitation (LOQ)

$$\text{LOQ}=10.0\times {\text{S}}_{\text{o}}/\text{b}$$

where S_O_ and b are standard deviation and slope of the calibration line, respectively.

## RESULTS

### Reaction with σ-acceptor

**Figure 2:**
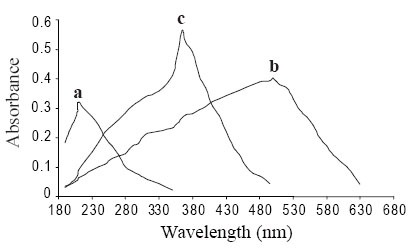
Absorption spectra of (a) 5.66 × 10 ^−3^ M Oxacillin sodium (50 μg/ml) in methanol (b) Blank Solution: 2.95 × 10 ^−1^ M iodine in dichloromethane (c) Sample Solution: 2.95 × 10 ^−1^ M iodine in dichloromethane + 1.81 × 10 ^−3^ M Oxacillin sodium (50 μg/ml) in methanol.

**Figure 3:**
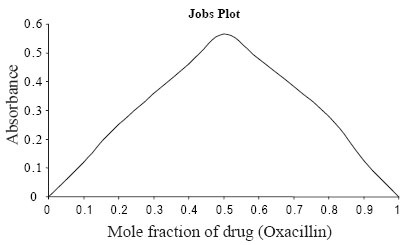
Plot of 1/D_0_ and [A_0_]/A^AD^ for the proposed method.

**Figure 4:**
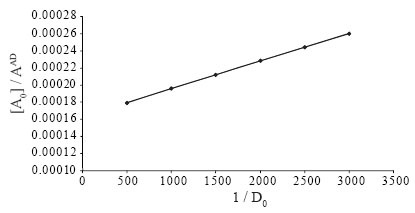
Jobs Plot for stoichiometric ratio between oxacillin sodium and Iodine (2.03 × 10 ^−3^ M each).

The absorption spectrum of iodine in dichloromethane showed only one peak with maximum absorption at 500 nm. The color of iodine changes to yellow upon reaction with Oxacillin sodium. This is due to charge transfer complexation reaction between Oxacillin with iodine. The absorption spectrum of oxacillin-iodine reaction product showed absorption peaks at 290 and 365 nm (Figure 2). The stoichiometry of the reaction was studied by Job’s method of continuous variations. It was observed from the Figure 3 that the combining molar ratio between oxacillin-iodine is 1:1. It has been reported that the charge-transfer complex between drug and iodine (11) would have an ionized structure DI^+^ ... I_3_^−^. The absorption spectrum of this charge-transfer complex is identical to that of I_3_^–^ in dichloromethane as it also absorbed at 290 nm and 360 nm. The association constant and apparent molar absorptivity of iodine-oxacillin sodium charge transfer complexes have been calculated using Ross and Labes equation (12), which depends on the experimental condition that acceptor concentration should not low enough to be considered negligible with respect to donor concentration.

(a) $$\frac{\left[\text{A}]\;[\text{D}\right]}{\left[\text{A}]+[\text{D}\right]}\times \frac{1}{{\text{A}}_{\lambda }}=\frac{1}{\text{K}{\text{ɛ}}_{\lambda }}\times \frac{1}{\left[\text{A}]+[\text{D}\right]}+\frac{1}{{\text{ɛ}}_{\lambda }}$$

where [A] and [D] are total concentrations of the acceptor and donor, respectively. A_λ_ and ɛ_λ_ are the absorbance and apparent molar absorptivity of the complex at wavelength. K is the association constant of the charge transfer complex.

$$\frac{\left[\text{A}]\;[\text{D}\right]}{\left[\text{A}]+[\text{D}\right]}\times \frac{1}{{\text{A}}_{\lambda }}\;\text{is}\;\text{plotted}\;\text{against}\;\frac{1}{\left[\text{A}]+[\text{D}\right]}$$

(Figure 4) which gave a straight line. The intercept and slope were calculated and equation (a) is transformed into the following equations:

(b) $$\frac{\left[\text{A}]\;[\text{D}\right]}{\left[\text{A}]+[\text{D}\right]}\times \frac{1}{{\text{A}}_{\lambda }}=3.202\times 10^{-7}\times \frac{1}{\left[\text{A}]+[\text{D}\right]}+1.641\times 10^{-3}$$

The association constants and molar absorptivities were found to be 3.826 ×10^−3^ and 1.203 × 10^−3^ l/mol/cm from equation (b) and the free energy change was also calculated and found to be –20.44 KJ/mol for Iodine-oxacillin complex.

## DISCUSSION

### Solution stability

The solution stability was ascertained from the UV spectra of the quality control samples. The complex showed an absorption peak at 365 nm. The quality control sample solutions were kept at room temperature for 10 days, and it was observed that there was no change in the absorption spectra of these solutions.

### Optimizations of variables

The different parameters affecting the color development were extensively studied to determine the optimum conditions for the assay procedures. The optimum values of the variables were maintained throughout the determination process.

### Effect of reaction time

The colored product was formed immediately and remained stable at room temperature for about 10 days. The absorbance was measured after 2 minute. of mixing the reagent.

### Effect of iodine concentration

**Figure 5:**
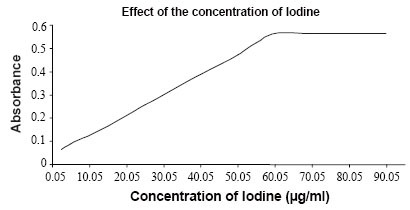
Effect of the volume of Iodine (0.05 %) on the absorbance; keeping constant 5.0 μg/ml Oxacillin sodium.

In order to study the effect of the volume of 0.05% iodine on the absorbance of the charge transfer complex, varying volumes (0.05–1.8 ml) were treated, separately with 8 μg of oxacillin. The results are shown in Figure 5 which indicated that 1.2 ml of 0.05% iodine gave the maximum absorbance and remained constant by further addition of iodine. Therefore, a volume of 1.5 ml was chosen for the determination of oxacillin sodium.

The robustness of the method was checked for each operational parameter and investigated. The operational parameters were also follows:

- Reaction time: 2 ± 1 Minute
- Reaction condition: Room Temperature ± 2°C

The robustness of the method was assessed by analyzing the Oxacillin Sodium. The quality control sample solution containing 5.0 μg/ml of the drug assayed. The percent recovery ± RSD of the method (99.985 ± 0.854) were found to be appreciable, indicating that the proposed method is robust.

For the evaluation of ruggedness of the proposed method, the contents of oxacillin at 5.0 μg/ml were assayed following the recommended procedure using UV 3000^+^ UV-VIS Spectrophotometer (LABINDIA^®^, Mumbai, India) and Chemito 2700 (New Delhi, India). The recoveries ± RSD resulting from the LABINDIA 3000^+^ (99.52 ± 0.429) and Chemito 2700 (99.46 ± 0.451) were compared.

### Analytical performance of the proposed method

**Figure 6:**
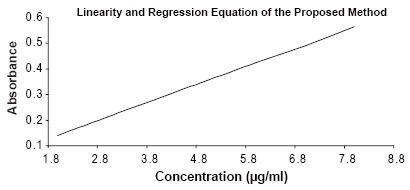
Linearity and linear regression equation of the proposed method.

Under the optimum experimental conditions, the absorbance-concentration plot for the proposed method was found to be rectilinear over the range of 2–8 μg/ml (Fig. 6). Linear regression analysis of calibration data gave the regression equation cited in Table 1 with correlation coefficient close to unity in both the cases. The suitability of the reaction products was estimated in the reaction mixture, it was found that the absorbance is stable for at least ten days at room temperature.

**Table 1:** Summary of optical and regression characteristics of the proposed method

Parameters	Oxacillin Sodium

Linear dynamic range (μg/ml)	2.0–8.0
Regression equation^a^	Y = 7.05 × 10^−2^*X*–7.0 × 10^−3^
S_a_	6.55 × 10^−4^
t S_a_ b	1.46 × 10^−3^
S_b_	1.41 × 10^−5^
t S_b_ b	3.13 × 10^−5^
Correlation coefficient (r)	0.9999
LOD (μg/ml)	3.90 × 10^−1^
LOQ (μg/ml)	1.18
Variance (S_o_^2^) of calibration line	1.04 × 10^−6^

a With respect to Y = a + b *X*, where X is the concentration in μg/ml, Y is Absorbance;

b Confidenceinterval of the intercept and slope at 95% confidence level and ten degrees of freedom (t=2.228).

The working standard sample solutions were stressed by light and heat (up to 50°C) for 5 hours. It was observed that stress by such conditions did not cause significant degradation. There is no change in the absorption spectra. The influence of frequently encountered excipients on the proposed method was studied by adding different amounts of possible interferents to the sample. It was observed that glucose, fructose, sucrose, cellulose, starch, lactose, magnesium stearate and sodium stearyl fumarate did not interfere the proposed method.

The accuracy and precision of the proposed method was established by performing intraday and interday assays by determining the content of Oxacillin samples at three different concentration levels (low, medium and high). These assays were investigated by measuring five independent analyses at 2, 5 & 8 μg/ml concentration levels within 1 day and on 5 consecutive days, respectively (Table 2). The standard deviation, relative standard deviation and standard analytical error obtained by both methods are acceptable i.e. within the permissible bias range and therefore can be considered to be satisfactory.

**Table 2:** Summary of accuracy and precision results of the proposed method in pure form

Proposed methods	Amount (μg/ml)		RSD %	REC.	SAE b	C.L.^c^
	Taken	Found ± SD^a^				

Intra day assay	2.00	1.999 ± 0.002	0.119	99.990	1.1 × 10^−3^	3.0 × 10^−3^
	5.00	4.999 ± 0.003	0.068	99.980	1.5 × 10^−3^	4.2 × 10^−3^
	8.00	7.999 ± 0.006	0.074	99.986	2.6 × 10^−3^	7.3 × 10^−3^
Inter day assay	2.00	1.999 ± 0.003	0.162	99.990	1.5 × 10^−3^	4.2 × 10^−3^
	5.00	4.997 ± 0.010	0.203	99.940	4.5 × 10^−3^	1.3× 10^−2^
	8.00	8.007 ± 0.014	0.175	100.085	6.3 × 10^−3^	1.7 × 10^−2^

a Mean for 5 independent analyses;

b SAE, standard analytical error;

c C.L., confidence limit at 95% confidence level and 4 degrees of freedom (t=2.776).

Recovery study was employed to check the validity of the proposed procedures. In this method, a known amount of excipients was added to its pure form at different concentration levels and the nominal value of the drug was calculated following the proposed procedure. The results are summarized in Table 3. As can be seen from the Table 3 that recovery obtained by the proposed procedure is quite satisfactory with low RSD. The results of the proposed method were statistically compared with those of the developed reference method (13) using point and interval hypothesis tests. Table 4 shows that the calculated (t-paired) and F-values at 95% confidence level are less than the theoretical ones, confirming no significant difference between the methods compared. It can also be seen from Table 4 that the bias evaluated by interval hypothesis test is within the acceptable range of θ_L_=0.98 and θ_U_=1.02.

**Table 3:** Summary of accuracy and precision results of the proposed method after adding excipients

Excipients	Amount (μg/ml)		RSD %	REC.	SAE^b^	C.L.^c^
	Taken	Found ± SD^a^				

Glucose	2.00	1.999 ± 0.004	0.210	99.980	0.0019	0.0053
Fructose	2.00	1.999 ± 0.011	0.569	99.970	0.0051	0.0142
Sucrose	2.00	1.999 ± 0.014	0.691	99.949	0.0062	0.0172
Cellulose	2.00	1.999 ± 0.010	0.512	99.946	0.0046	0.0128
Starch	2.00	2.000 ± 0.005	0.232	100.02	0.0021	0.0058
Lactose	2.00	1.999 ± 0.017	0.851	99.949	0.0076	0.0211
Magnesium Stearate	2.00	1.999 ± 0.012	0.601	99.927	0.0054	0.0150
Sodium Stearyl Fumarate	2.00	1.999 ± 0.009	0.464	99.970	0.0042	0.0117

**Table 4:** Summary of comparison results of the proposed method with reference method (13) at 95% confidence level

Parameters	Proposed method	Reference method (13)

Recovery (%)	99.987	99.938
RSD	0.245	0.480
t	0.51	
F	2.85	
θ_L_	0.985	
θ_U_	1.008	

The proposed method was further extended to the *in vitro* determination of Oxacillin in human urine samples. The results are summarized in Table 5. These results are satisfactorily accurate and precise because it gives higher recovery and low relative standard deviation.

**Table 5:** Application of the proposed method to the determination of Oxacillin sodium with spiked human urine samples

Amount added (μg/ml)	Amount found (μg/ml)	Recovery (%)

2.0	1.9412	97.06
3.0	2.9286	97.62
4.0	3.9257	98.14
5.0	4.9176	98.35
6.0	5.9093	98.49
8.0	7.8919	98.65
X		98.05
RSD		0.615

## CONCLUSIONS

The proposed developed method is a spectrophotometric method requiring only single reagent iodine for its colour development in methanol-dichloromethane. The method does not require any laborious clean up procedure before measurement. In addition, the method has wider linear dynamic range with good accuracy and precision. The recovery results obtained is quiet satisfactory with low relative standard deviation. The method shows no interference from the common excipients and additives. The proposed method was compared with the reference method which indicate good recovery with θ_L_=0.985 and θ_U_=1.008. The proposed method is not time consuming; do not involve any pretreatment of samples. In HPLC and other reported method requires more time and costly solvent and column. The solution stability of the quality control samples of the proposed method is more than the other reported methods. Ultimately the UV-VIS spectrophotometry is low cost techniques and it is easily available in any where. Therefore, it is concluded that the proposed method is simple, sensitive, accurate and rapid for the determination of Oxacillin in bulk and in human urine samples.
